# Cancer Genomics

**DOI:** 10.12688/f1000research.6645.1

**Published:** 2015-10-28

**Authors:** Elaine Mardis

**Affiliations:** 1 McDonnell Genome Institute, Washington University School of Medicine, Campus Box 8501, 4444 Forest Park Avenue, St. Louis, MO, 63108, USA

**Keywords:** cancer, genomics

## Abstract

Modern cancer genomics has emerged from the combination of the Human Genome Reference, massively parallel sequencing, and the comparison of tumor to normal DNA sequences, revealing novel insights into the cancer genome and its amazing diversity. Recent developments in applying our knowledge of cancer genomics have focused on the utility of these data for clinical applications. The emergent results of this translation into the clinical setting already are changing the clinical care and monitoring of cancer patients.

## Introduction

Even before we knew of DNA’s role in determining cellular function and biology, even before we knew chromosomes were made of DNA, there was speculation that the source of cancer somehow was determined by profound changes in the chromosomes ^1^. Early pioneers in cancer genomics, such as Janet Rowley (cited in this review), provided substantial evidence of a role for the genome in cancer’s development by observing microscopically that patients with specific subtypes of leukemia shared specifically altered chromosomes ^2– 4^. Initially, these chromosomal translocations were used to provide diagnostic evidence of the specific subtype, and as our characterization of these translocations became more precise, the fusion gene drivers of oncogenesis such as BCR-ABL and PML-RARα were identified and defined according to their mechanisms. Ultimately, several of the recurrent genomic events in hematologic malignancies have been targeted by highly specific and effective therapies, rendering them manageable from a clinical standpoint and permitting patients either to survive cancer as a chronic disease, especially with the development of specific second- and third-line therapies that address acquired resistance mutations in the targeted fusion proteins, or to be cured outright (for example, approximately 94% of patients with acute promyelocytic leukemia are cured by all-trans retinoic acid or arsenic consolidation therapies).

As the Human Genome Project drew to a close in the early 2000s, scientists had a template or keystone with which they could compare and characterize changes to the genome in disease states such as cancer ^5^. Initially, however, sequencing technology did not permit the sequencing of the entire genome at reasonable cost and throughput, so several groups began to design pipelines for high-throughput polymerase chain reaction (PCR) amplification and sequencing of known cancer genes in an effort to catalogue cancer-specific (“somatic”) mutations. During this same time frame, pharmaceutical companies began to perform clinical trials of drugs for solid tissue malignancies in major cancer centers that targeted specific proteins or protein families thought to be drivers of oncogenesis. In some but not all cases, these tyrosine kinase inhibitors (TKIs) were highly successful at achieving dramatic reductions of tumor burden in some (but not all) advanced metastatic patients. Given these remarkable results and the differential patient responses, focused efforts began to identify whether specific mutations could be correlated with response. In 2004, three groups published independently that, in non-small cell lung adenocarcinomas (NSCLCs), approximately 80% of responders to TKI therapy could be correlated with patients having mutations in the tyrosine kinase domain of the epidermal growth factor receptor (EGFR) ^6– 8^. As remarkable as these responses were, patients frequently relapsed, often with more aggressive and widespread disease after several months of treatment. As initially defined by Engelman and colleagues, these examples of acquired resistance to targeted therapy were due to new mutations in EGFR that conferred a lack of response to the TKIs because of reduced binding affinity ^9^.

In the midst of these efforts to catalogue the mutations in cancer genes, transformative sequencing technologies were emerging. So-called “massively parallel” sequencing (MPS) technologies, they coupled the molecular biology of polymerase-catalyzed sequencing with light-based detection to report the incorporated nucleotides for each of several hundred thousand sequencing reactions taking place simultaneously ^10, 11^. These technologies further streamlined the sequencing library preparation steps and permitted pooled PCR products to be sequenced in the same instrument run, thereby accelerating throughput, reducing sequencing costs, and introducing a “digital” type of data that sequenced individual DNA molecules (after *in situ* amplification). Although these platforms introduced new challenges into data analysis based on the initially short reads relative to capillary sequencers, early efforts ^12– 15^ defined methods for whole genome sequencing of tumor and normal genomes and their comparison in order to identify somatic mutations in an unbiased way. A “middle ground” between directed PCR of genes and whole genome sequencing was developed and reported by several groups to capture by hybridization the exonic portion of the genome (“exome”), providing a more conscripted yet easier to analyze and interpret subset of the genome ^16– 18^. What has followed during the time period from around 2009 to the present is large-scale discovery, by MPS-based methods, of somatic alterations in thousands of cancer genomes, including comparisons of the tissue site-specific range and diversity of mutational load genome-wide ^19^, the identification of phenomena such as chromothripsis ^20^ and kataegis ^21^, and a broad-based recognition that cancer genomes find myriad and different ways to create themselves.

Several early studies pioneered the notion of using high-depth digital MPS-based sequencing and clustering of mutation sites with shared variant fractions of reads to evaluate the changes in clonal heterogeneity that occur between primary and metastatic or recurrent disease ^22, 23^. Recent comparisons of this type have explored changes to the cancer genome in the transition from treatment-naïve to post-therapy recurrent disease ^24– 29^. One challenge that has limited these types of studies in solid tissue malignancies has been the difficulty in obtaining post-treatment biopsies, which often cannot be obtained as the standard of care and/or may have associated risk or morbidities.

Given the range and scope of discovery that have taken place over the past five years, a basic understanding of the tumor genome landscape has been defined for most of the prevalent tumor types and a few rare ones as well. There is ample evidence that, given this body of knowledge and pertinent clinical questions that may be further informed by genomics, the clinical translation of genomics is an obvious next step. This review will focus on three pertinent aspects of clinical translation for cancer genomics in an effort to highlight the trends and add evidence from the existing body of translational work that genomics already is impacting and will continue to impact on cancer medicine.

## Tumor evolution and changes in genomic heterogeneity

Several groups have built upon early studies and methods that evaluated deep coverage at mutation sites to build models of founder and subclonal cell population genotypes. As mentioned, recent studies have focused on the comparison of primary with metastatic or of treatment-naïve with recurrent post-treatment tumors. The comparisons of primary with metastatic disease in solid tissue malignancies have illustrated the persistence of the founder or trunk mutations into metastases, with new mutations being acquired in different metastatic sites. These studies ^30– 32^ build upon, but somewhat differ in their conclusions when compared with, the earlier work by Gerlinger and colleagues ^33^, who reported comparisons of primary with metastatic renal cell carcinomas.

Similar studies have evaluated treatment-naïve to recurrent disease in the setting of DNA-damaging chemotherapy, establishing a mutational “signature” in the recurrent disease setting that defines the resulting DNA damage and results in an elevated mutation rate. Our early work describing this result in recurrent acute myeloid leukemias ^22^ was recently followed by a study of post-temozolomide-treated pediatric gliomas, illustrating a profound increase in the number of mutations from exome sequencing-based comparisons ^34^. In both cases, the emergent disease has a mutational landscape akin to carcinogen-associated mutational processes, such as those observed in lung cancer due to smoking or in melanomas due to ultraviolet (UV) exposure. Another study of platinum-resistant high-grade serous ovarian cancer has identified post-therapy resistance signatures akin to BRCA (breast cancer, early onset)-associated mismatch repair (MMR) defects ^35^ or, in a minority of samples, the apolipoprotein B mRNA editing enzyme-related (APOBEC) defects ^36^. However, the predominant impact in high-grade serous ovarian disease for platinum resistance appears to be due to gene breakage defects at tumor suppressor loci, discernable only by the integration of whole genome and transcriptome data performed in this study ^35^. Interestingly, the sequencing results revealed a higher mutational burden measured as single-nucleotide variants and insertion-deletion variants when comparing the platinum-resistant recurrent tumor cells derived from ascites fluid with the primary tumor. A significant relationship between the number of non-coding mutations and the numbers of courses of platinum-based chemotherapy the patient received also was described.

Recent genomic comparisons of matched treatment-naïve disease with post-therapy recurrent tumors have mainly studied patients emerging with acquired resistance to targeted therapy treatment ^24, 37, 38^. The results have elucidated the nature and types of mutations that are conferring therapy resistance and give rise to the hope that pinpointing the genomic source(s) of acquired resistance to targeted therapies might be more straightforward and less complicated than to chemotherapies. In one report regarding the genomics of therapy-resistant EGFR-mutated NSCLCs, the mechanism for a rarely observed transition of NSCLCs into small cell lung carcinoma was elucidated as being due to loss of RB1, solving a long-standing puzzle ^39^.

Therefore, it is important to understand the genomic alterations that might lead to treatment resistance, where possible. When these alterations are identified as the means by which the tumor cells can evade the mechanism of therapeutic action, real-time blood-based monitoring for the rise and fall of the acquired resistance alteration(s) may be possible. This genomics application addresses the difficulty of obtaining recurrent tumor biopsy material for genomic testing. Often, the sensitivity of blood-based monitoring or “liquid biopsy” over imaging-based detection of recurrent tumor growth is quite desirable as well. In the next section, the concepts and practices of liquid biopsy will be addressed as a means of introducing this alternative approach to tumor progression and treatment response monitoring.

## Liquid biopsy

Although some of the cancer genomics discovery work that was discussed above has contributed substantially to our understanding of the genomic relationships between primary and metastatic disease in the same patient, the reality is that obtaining a metastatic resection or biopsy sample is often not the standard of care and therefore is not reimbursable by private insurance payors. Beyond these practical considerations, metastases can be inaccessible and therefore difficult to sample. Small studies of multiple metastatic lesions have indicated that there are differences in the genomes of metastases in different sites that must be considered in tracking the progression or stability of the cancers present in the individual. There also can be associated morbidity and risk with biopsy procedures that diminish the enthusiasm of study participants to undergo the procedure. Suffice it to say that a proxy for detecting solid tumor progression-associated changes is badly needed in cancer medicine.

In this regard, an opportunity may be present in assays referred to collectively as “liquid” or blood-based biopsy, whereby a blood sample is obtained from a patient at diagnosis and compared with sequential temporal blood samples obtained during treatment for the purposes of monitoring tumor burden, often as a function of response to therapy ^40– 45^. From these blood samples, one can study the DNA shed from tumors as cells turn over, in the form of mutation-specific assays of circulating free DNA (cfDNA), or DNA from isolated circulating tumor cells (CTCs) or from tumor-derived exosomes.

Each approach has its own nuances, including specialized isolation approaches and assay types, as follows. CTCs are rare cell types that can be isolated from the blood and indeed may fluctuate in their prevalence and representation of the mutational landscape according to disease stage, tissue site, and other factors required for isolation such as cell surface markers ^46– 48^. Typically CTCs require specialized instrumentation to isolate, of which several types are available commercially, any one of which may be more applicable to different tumor types. Also, the rarity of CTCs requires higher amounts of blood input, which can impose a practical/clinical limitation. After isolation and cell lysis, whole genome amplification of CTC DNA is followed by whole genome, exome, or targeted sequencing. cfDNA, by contrast, requires isolation from plasma within a few hours of blood draw to minimize degradation and varies in amount according to disease stage and tissue site. Mutational assay of cfDNA requires focused PCR of known or suspect mutations, due to the degraded state of tumor DNA in the circulation, followed by high-depth sequencing to overcome the background of cfDNA provided by normal cell apoptosis ^49– 51^. Exosomes, which are small (950–1000 μm) vesicles containing DNA, RNA, and protein components from apoptotic tumor cells, also are shed at lower amounts by normal cells. Owing to the contents of exosomes, evaluation may occur by multiple assay types to identify DNA, RNA, or protein related to tumor monitoring. There are several different isolation procedures for obtaining purified exosomes from blood, ranging from low-throughput differential ultracentrifugation to size- or affinity-based purification ^52^.

Regardless of the type of blood biopsy, there is increasing evidence that this approach will be broadly applicable to monitoring patient response to neo-adjuvant therapy, to surgery, or to surgery followed by chemo-, radiation, or targeted therapy. With the genomic characterization of acquired resistance mutations arising in the targeted therapy setting, precise mutational analyses can detect patients who are developing acquired resistance in a much more sensitive way than by conventional imaging, which can often be misleading regarding objective response to a therapeutic intervention ^51^. Depending upon the approach, blood-based monitoring also is quite rapid and inexpensive relative to imaging, yet more studies are required to fully understand its applicability and limitations.

## Immunogenomics

Immunogenomics is a somewhat broad term that refers to numerous genomics-based inquiries that (1) may assay specific immune components in their interaction with established cancers, (2) may indicate the likelihood of a tumor to respond to immunotherapy, or (3) may be used to design personalized vaccines for individual patients deemed likely to respond to an immune modulatory therapy. Much of the foundational work in immunogenomics stems from studies of melanoma, a tumor type long recognized as having extensive immune system interactions ^53, 54^. Sequencing of DNA isolated from melanomas has defined the signature of UV-associated DNA damage ^55^ and has identified that melanomas have overall one of the highest mutation rates of any tumor type, as a result of UV damage ^19, 56^. In 2010, the first results of clinical trials in melanoma testing a new class of immunotherapeutic, called “checkpoint blockade immunotherapy”, were announced, showing dramatic responses in some advanced metastatic patients ^57^. In 2011, the US Food and Drug Administration (FDA) approved the use of anti-CTLA4 immunotherapy (ipilimumab or Yervoy™; Bristol-Myers Squibb Company, New York, NY, USA) for the treatment of metastatic melanoma. Subsequent FDA approvals have been granted for immunotherapies targeting another checkpoint blockade protein, PD1, in melanoma (nivolumab and pembrolizumab). These therapies have expanded into single-agent clinical trials of other cancer sites, including non-small cell lung and bladder cancers, and also are showing significant response rates when used in combination ^58– 60^. Nivolumab was recently approved by the FDA for previously treated advanced or metastatic NSCLCs. Like melanomas, these tumors are associated with the carcinogens in cigarette smoke and have a correspondingly high mutation rate across the genome. Whether combination checkpoint blockade therapies in smoker-associated lung adenocarcinomas will have increased efficacy as seen in melanomas remains to be tested.

Studies of mouse models of sarcomas induced by a chemical carcinogen, methylcholanthrine (MCA), have been used to study the interaction between the immune system and cancer ^61^. A genomic study of these mouse model tumors revealed an MCA-specific mutational signature and a high mutational load. Combined exome sequencing with neoantigen prediction algorithms (based on major histocompatibility complex [MHC] binding avidity comparing mutated to wild-type peptides) identified those tumor-specific mutant antigens (TSMA) or “neoantigens” that were specifically targeted by the immune system to effect elimination of growing tumors ^62^. More recently, this MCA model and the same genomics-based approach were used to demonstrate that TSMA were also the proteins targeted by anti-CTLA4 or anti-PD1 antibodies, and importantly that synthetic peptides corresponding to TMSA could be used as a prophylactic or therapeutic vaccine ^63^.

In human cancers, exome sequencing and neoantigen prediction have now characterized that patients with melanoma who responded to anti-CTLA4 checkpoint blockade have a high number of non-synonymous mutations ^64^. Similar results were described on the basis of only exome sequencing data for lung cancer patients with anti-PD1 responses ^65^, for bladder cancer and other high mutational load cancers with anti-PD-L1 responses ^66, 67^, and recently for MMR-deficient colon and other MMR-deficient cancers treated with anti-PD1 therapy ^68^. These results, though exciting, raise the issue of whether this high mutation rate is a biomarker of sorts for gauging which patients will respond to these therapies. Likely, it is more complicated since even in the small number of MMR-deficient patients who received anti-PD1 therapy, there were a small number of non-responders. To state the question in another way, will all tumors with a significant mutational load respond to checkpoint blockade? Or should the mutations be further evaluated algorithmically for their antigenic potential as neoantigens? How does the predictive quality of mutational load characterization or neoantigen load compare with immunohistochemistry-based evaluation of PD1 and PD-L1 protein expression? These open questions require further study and the requisite comparisons of predictive power. Regardless of the answers, the notion that the mutational load of non-synonymous mutations in the tumor exome can predict therapeutic response fundamentally changes our definition of an “actionable mutation”.

Using an analytical approach similar to that described above for the MCA mouse models to predict neoantigens, we combined exome sequencing with algorithmic prediction of MHC binding to compare tumor-unique peptides with their wild-type counterparts in a small clinical trial of patients with melanoma. This approach identified the neoantigens most likely to stimulate tumor-specific T cells, which were further evaluated for RNA expression of the mutant alleles and then evaluated with patient-derived immune components *in vitro*. The neoantigenic peptides were synthesized and used to condition patient-derived dendritic cells to create personalized vaccines for three patients ^69^. In all three patients receiving vaccines and post-vaccine monitoring to date, three of the seven tumor-specific peptides elicited a T-cell response that was measurable after vaccination. In determining the neoantigens to include in each patient’s vaccine, we evaluated inter-metastatic heterogeneity by producing exome sequencing from multiple biopsies in two of the three patients. We also used T-cell receptor-specific PCR and MPS to characterize the resulting T-cell repertoire from blood. Here, we determined that for the three peptides eliciting an enhanced T-cell expansion in each patient, the T-cell receptor repertoire was very diverse, representing multiple clonotypes. These studies demonstrate how cancer genomics-based approaches are being used to characterize the mutational load of tumors that do or do not respond to checkpoint blockade immunotherapies or to design personalized immunotherapies and monitor the resulting T-cell repertoire in vaccinated patients.

## Future forward

Cancer genomics has progressed dramatically in its application to clinical questions of cancer care in just a few short years. This translational trajectory has been demonstrated in several ways. Firstly, the use of deep sequencing and analysis to evaluate the evolution of cancers via clonal heterogeneity changes has revealed important information about the nature of acquired resistance to targeted therapies and chemotherapies. Secondly, the concept of tracking emerging resistance to therapy has led to the notion of blood-based monitoring via “liquid biopsy” as a sensitive and inexpensive proxy for tumor response. Thirdly, a surprising application for cancer genomics has emerged from studies of the immune system’s interaction with cancer, supporting the notion that mutational load via genomics may be a predictor of response to checkpoint blockade therapy. Importantly, if mutational or neoantigen load is a predictor of checkpoint blockade response, we may, in our clinical use of DNA-damaging chemotherapy as the standard of care for many patients, be creating an opportunity to use immunotherapies as a second-line therapeutic approach. This is predicted by genomic studies of post-therapy recurrent tumors or metastases that indicate a signature of DNA damage and a correspondingly higher mutation rate resulting from DNA-damaging chemotherapies ^22, 34^.

Genomics also is contributing to personalized vaccine development efforts by identifying tumor-specific neoantigens that potentially can stimulate T-cell memory against cancer cells. Though still in development, the vaccine “angle” provided by genomics may provide an important possibility to cancer patients who have exhausted other treatment approaches, including other types of immunotherapy. Although cancer remains a significant and as-yet-unsolved disease, modern cancer genomics is contributing to clinical diagnosis and to therapeutic decision-making. Taken together, impactful clinical translational efforts involving cancer genomics should continue for some time to come. It will be exciting to see the results!
